# Supplementary material for: Physical Properties of Thermally Crosslinked Fluorinated Polyimide and Its Application to a Liquid Crystal Alignment Layer
Source: Polymers (Basel). 2021 Nov 11;13(22):3903. doi: 10.3390/polym13223903 (PMC8621600; doi:10.3390/polym13223903)
Supplement: Supplementary file 1 [file polymers-13-03903-s001.zip › polymers-1429664-supplementary.pdf]

Supplementary Materials

Physical Properties of Thermally Crosslinked Fluorinated Polyimide and Its Application to a Liquid Crystal Alignment Layer

Jong-Soo Ahn,[1] Su Hong Park,[1] Na Yeon Kwon,[1] Min Ju Cho,[1,*] Sang-Hyon Paek,[2,*] Dong Hoon Choi[1,*]

[1]Department of Chemistry, Research Institute for Natural Science, Korea University
145 Anam-ro, Seongbuk-gu, Seoul 02841, Republic of Korea
[2]Department of Chemical Engineering, Kyung Hee University
1732, Deogyeong-daero, Giheung-gu, Yongin-si, Gyeonggi-do 17104, Republic of Korea

*Author to whom correspondence should be addressed.
E-mail: chominju@korea.ac.kr, shpaek@khu.ac.kr, dhchoi8803@korea.ac.kr

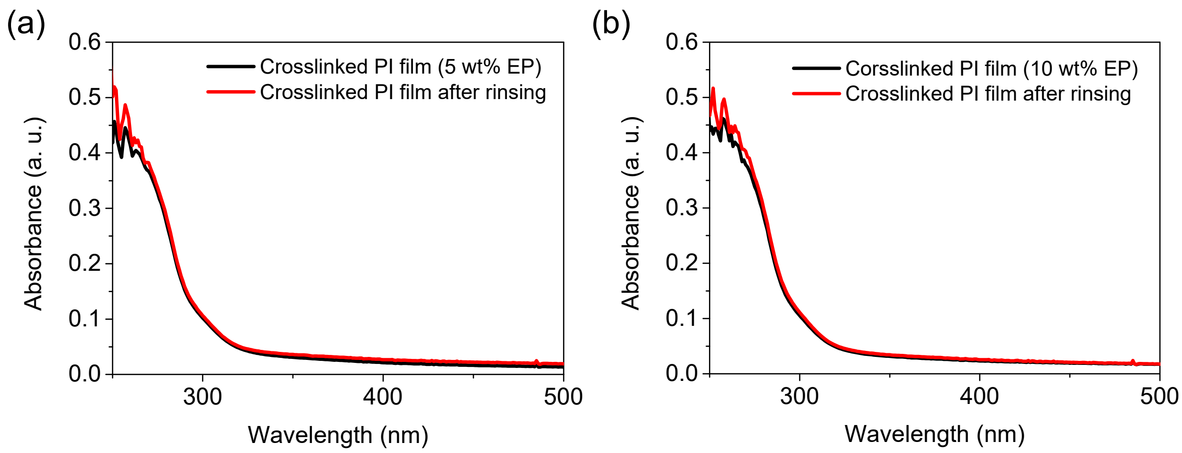

Figure S1. Absorption spectra of crosslinked PI obtained before and after rinsing (1 min) in THF. (a) 5 wt% EP and (b) 10 wt% EP.

The crosslinking reaction of 6FDA-ODA-based soluble PI was induced while increasing the concentration of EP from 5% to 10%. As a result, sufficient solvent resistance and heat resistance could be ensured even in the case of PI containing 5% EP as shown in **Figure S1**. Therefore, in the preparation of the crosslinked PI produced in this study, the entire study was conducted by mixing 5% EP and the corresponding PAA.

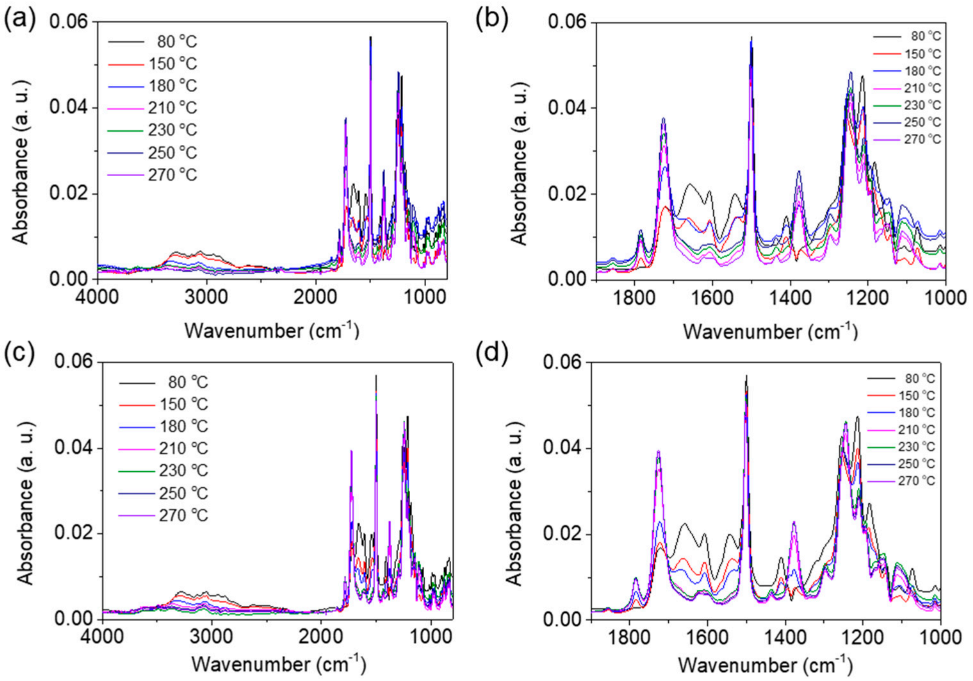

Figure S2. ATR-FTIR spectra of 6FDA-ODA PAA and 6FDA-ODA PAA with 5% EP during thermal treatment at an elevated temperature.

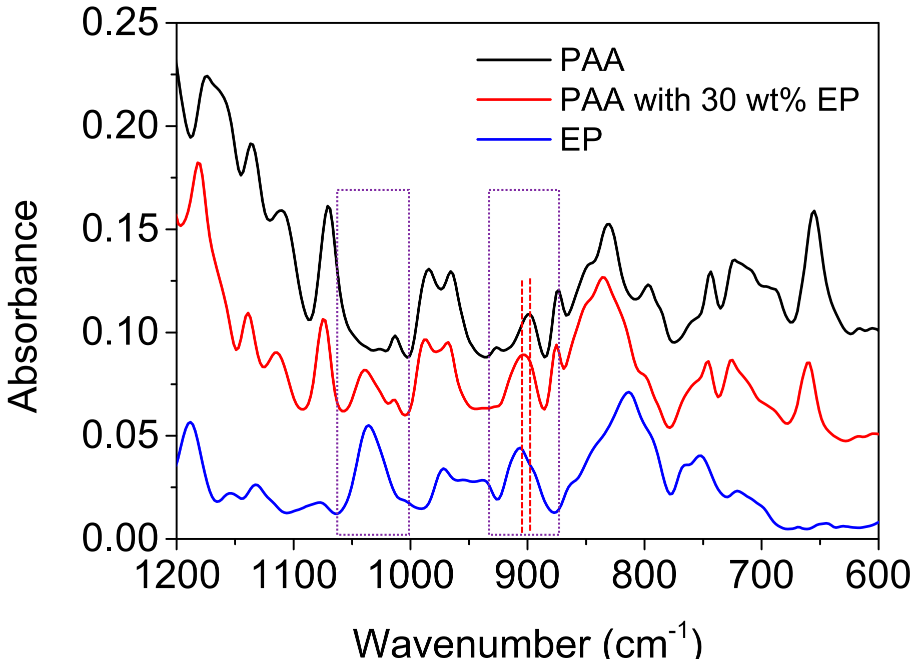

Figure S3. FT-IR spectra of PAA, PAA with 30 wt% EP, and EP samples.

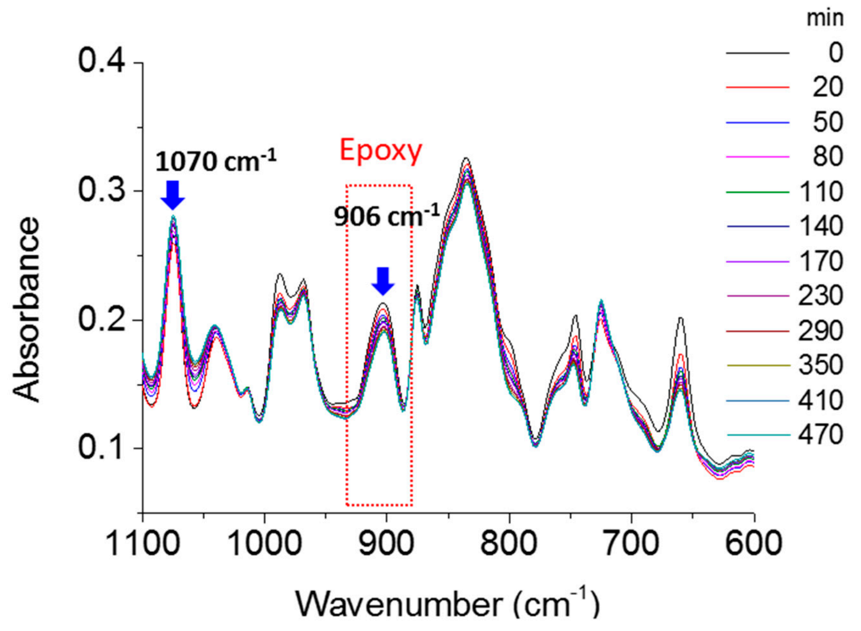

Figure S4. FT-IR spectra of a sample bearing PAA and 30 wt% EP subjected to heat treatment at different times at 50 °C.

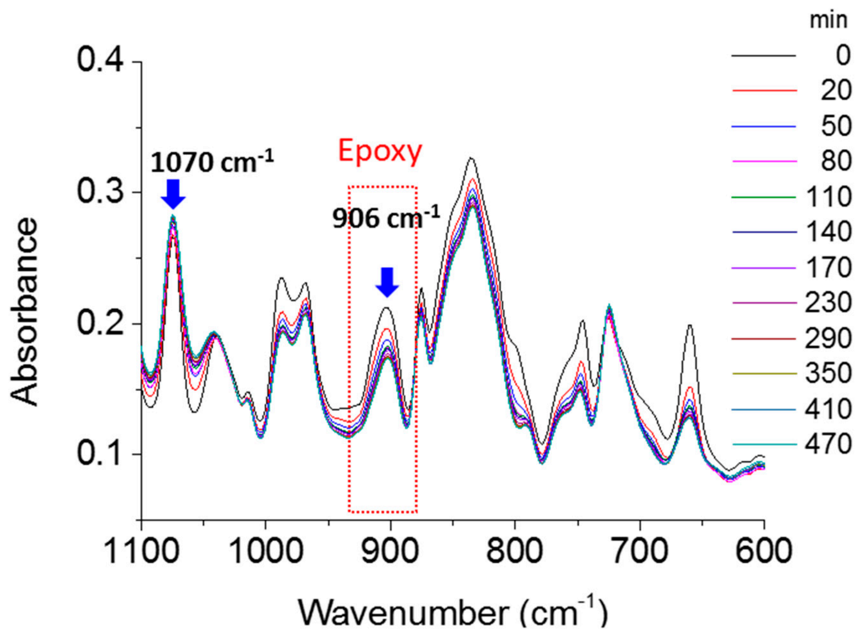

Figure S5. FT-IR spectra of a sample bearing PAA and 30 wt% EP subjected to heat treatment at different times at 70 °C.

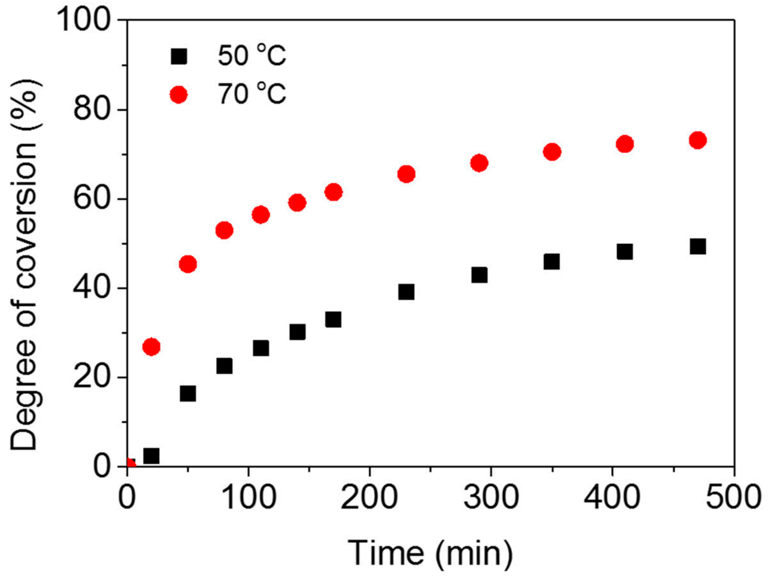

Figure S6. Degree of conversion over time for two different heat treatments using PAA with 30 wt% EP sample.

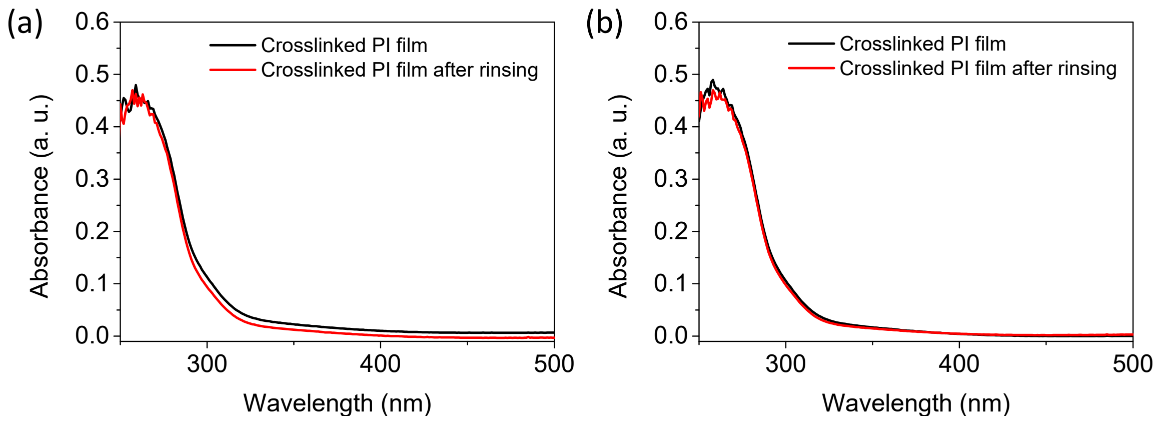

Figure S7. Absorption spectra of crosslinked PI obtained before and after rinsing (1 min) in DMF (a) and NMP (b).

We also performed solubility tests of the crosslinked PI in DMF and NMP solvents and the results were shown in **Figure S7.** The crosslinked PI was insoluble in DMF and NMP solvents.

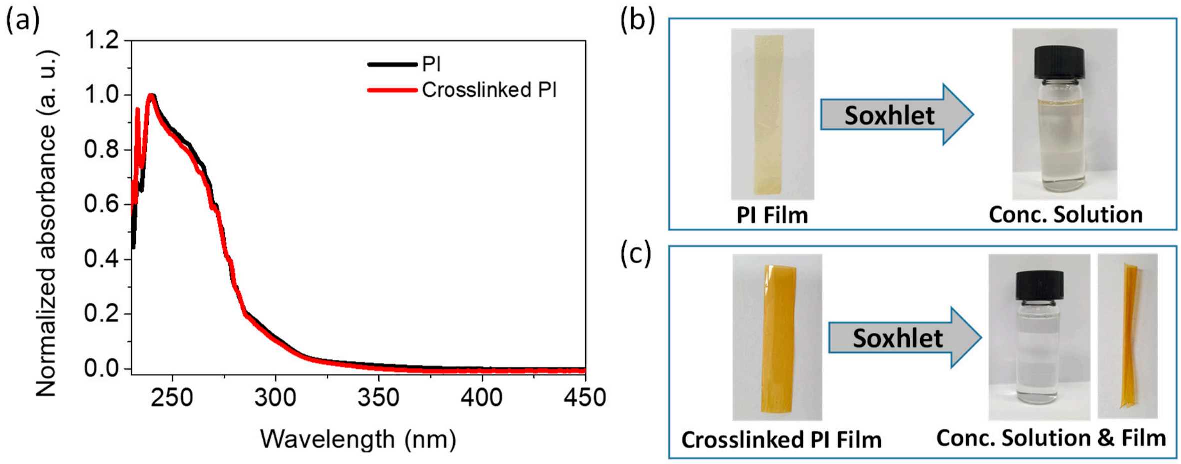

Figure S8. (a) The absorption spectrum of the THF solution of neat PI film and the absorption spectrum of the extracted solution obtained after Soxhlet extraction of crosslinked PI using THF solvent. (b) Image of neat PI film and its THF solution. (c) Crosslinked PI film, its extracted THF solution and undissolved crosslinked PI film.

In the case of the neat PI film during the Soxhlet extraction process, it was completely dissolved in THF (**Figure S8b**), and in the crosslinked PI film, a very small amount of non-crosslinked PI was dissolved. (**Figure S8c**). As a result of measuring the absorption spectrum using the extracted solution, it was confirmed that the dissolved portion contained neat PI. (**Figure S8a**) On the other hand, as shown in **Figure S8c**, the extract is almost colorless, and shrinkage and warpage of the crosslinked PI film are observed due to the dissolution of the uncross-linked part. And as a result of measuring the initial weight of the crosslinked PI film and measuring the weight of the dried PI film after Soxhlet was completed, a weight reduction of about 8% occurred.

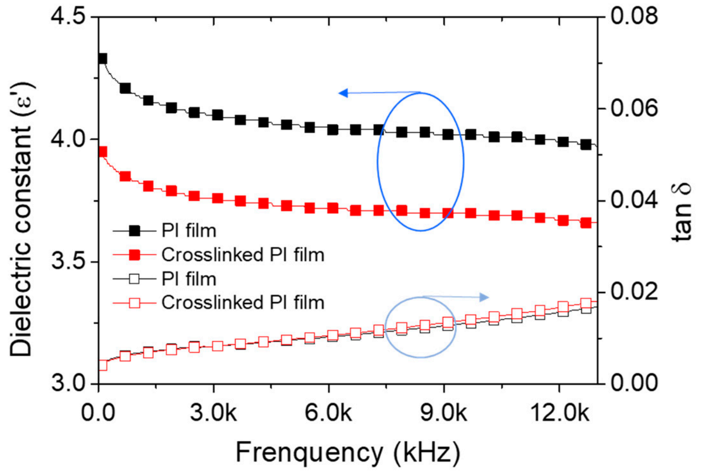

Figure S9. Frequency dependence of dielectric constants of neat 6FDA-ODA PI film and crosslinked PI film.

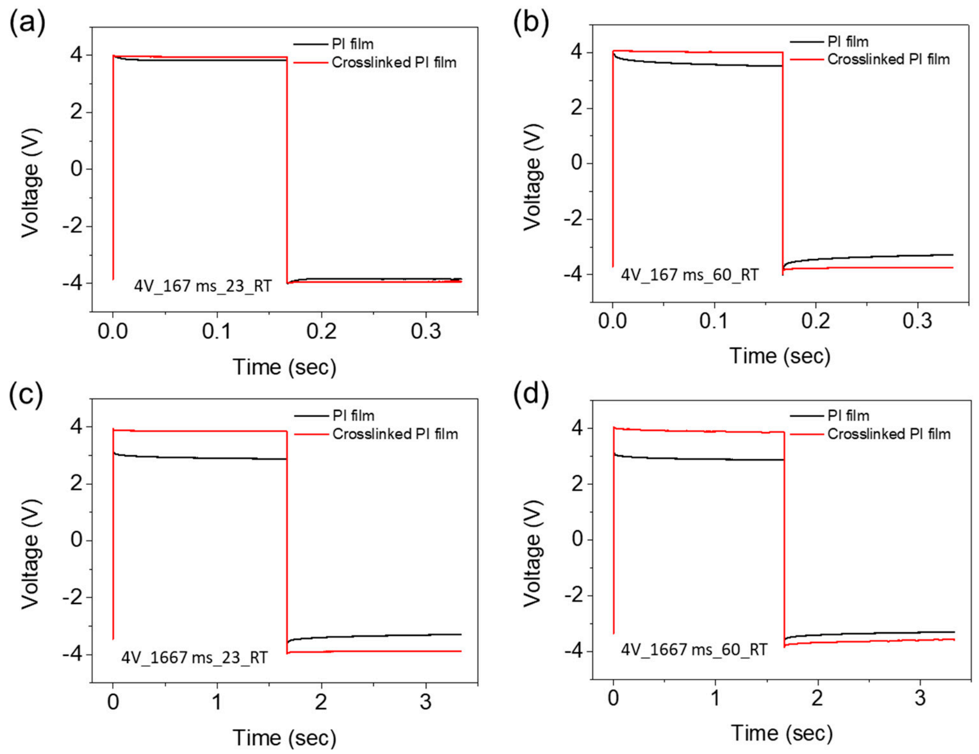

Figure S10. Voltage holding ratio (VHR) plots of the LC cells fabricated with the 6FDA-ODA PI AL and its crosslinked PI AL.
